# Treatment of Hernia in Children

**Published:** 1890-02

**Authors:** A. Jacobi

**Affiliations:** New York


### TREATMENT OF HERNIA IN CHILDREN.

BY A. JACOBI, M. D., NEW YORK.

*Archiv. Pediat.*, October: Umbilical hernia is of very frequent occurrence, but seldom attended with danger. Incarceration takes place very rarely; still, Treves and others have reported successful operations for such accidents. As there is a predisposition to the development of this variety of hernia, so there is a tendency toward spontaneous recovery. The round umbilical aperture will normally change after a number of months, or even a year, into a narrow fissure, more fat will develop, the muscles will become stronger, and then the intestine will be retained within the abdominal cavity. To accomplish this still more certainly, it is desirable to retain the contents of the hernia sac inside the abdomen. For this purpose, trusses are very unavailing. Strips of adhesive plaster will serve very much better, but in most cases they are objectionable because they irritate the sensitive skin of the baby.

Whatever application is made to the hernia directly, must be larger than the aperture. It should not be too hard. Linen compresses, and those of woven lint, plates of cork covered with linen or lint may be applied and held in position by means of a bandage. Knitted bandages will suit better than the ordinary bandage of linen, cotton or flannel.

Inguinal hernia in the newly-born or the very young, is apt to recover spontaneously. When the short and straight inguinal canal becomes longer and more oblique, in the course of a few years, and the amount of fat goes on increasing, the rupture may disappear; but all these predisposing factors never succeeded in making a cure by themselves. This was accomplished only when the hernia was retained inside the abdominal cavity completely and constantly, by means of a truss, which may be worn for years. It must not be removed except when the baby is sleeping quietly. Trusses are uncomfortable in the beginning, and give rise to cutaneous irritation, particularly under the influence of urine. So much the more is it necessary to keep the truss clean, and always to select one which fits exactly without exerting too much pressure.

Hernia is easily reduced into the abdominal cavity. But there are on record a number of cases in which incarceration and strangulation required operative interference. The operation should not be delayed after reduction proved impossible, even under the influence of an anæsthetic. An instance of a successful operation on a case of strangulated femoral hernia, which occurred in a girl of eleven years, has been reported by St. Germain. Rees succeeded in reducing an inguinal hernia by aspirating from the intestine a quantity of turbid liquid. An exceptional case of the kind, however, must not be recommended for general adoption.—*The Epitome.*

